# Molecular mechanisms of apoptosis suppression by HTLV-1 bZIP factor in HTLV-1 infected cells

**DOI:** 10.1186/1742-4690-11-S1-O51

**Published:** 2014-01-07

**Authors:** Azusa Tanaka-Nakanishi, Jun-ichirou Yasunaga, Ken Takai, Masao Matsuoka

**Affiliations:** 1 Laboratory of Virus Control, Institute for Virus Research, Kyoto University, Sakyo-ku, Kyoto, Japan

## 

HTLV-1 is linked to adult T cell leukemia (ATL) and several chronic inflammatory diseases, such as HTLV-1 associated myelopathy (HAM). The minus strand of HTLV-1 provirus encodes HTLV-1 bZIP factor (HBZ), which is expressed in all ATL cells and a key player in the proliferation of infected cells. One of the hallmarks of cancer cells is a property to escape from apoptosis. The aim of this study is to clarify the effects of HBZ on apoptosis in HTLV-1-infected cells. There are two major pathways for apoptosis: the extrinsic and intrinsic apoptotic pathways, which are mediated by Fas and Bim respectively. We analyzed the transcriptional profile of HBZ expressing cells, and found that the transcription of pro-apoptotic genes, Bim and Fas ligand (FasL), were both suppressed by HBZ. HBZ physically interacts with FoxO3a, a critical transcription factor of Bim and FasL genes, and attenuates the DNA binding ability of FoxO3a. In addition, HBZ induces the mislocalization of inactive phosphorylated FoxO3a in the nucleus, whereas this form exists in the cytoplasm in HTLV-1-negative cells. Besides of FoxO3a perturbation by HBZ, we have identified the epigenetic aberrations, DNA methylation and histone modification, in the promoter region of the Bim gene in ATL cells. The suppression of pro-apoptotic genes, Bim and FasL, by HBZ results in the reduction of activation-induced apoptosis. These results suggest that the attenuating FoxO3a function by HBZ may play an important role in increasing the number of infected cells and might be associated with HTLV-1-induced inflammation and leukemogenesis.

